# Long-read genome assembly and gene model annotations for the rodent malaria parasite *Plasmodium yoelii* 17XNL

**DOI:** 10.1016/j.jbc.2023.104871

**Published:** 2023-05-27

**Authors:** Mitchell J. Godin, Aswathy Sebastian, Istvan Albert, Scott E. Lindner

**Affiliations:** 1 Department of Biochemistry and Molecular Biology, The Huck Center for Malaria Research, The Center for Eukaryotic Gene Regulation, Pennsylvania State University, University Park, Pennsylvania, USA; 2 Huck Institutes of the Life Sciences, Pennsylvania State University, University Park, Pennsylvania, USA

**Keywords:** *Plasmodium yoelii* 17XNL, malaria, genome assembly, gene models, long-read sequencing

## Abstract

Malaria causes >600 thousand fatalities each year, with most cases attributed to the human-infectious *Plasmodium falciparum* species. Many rodent-infectious *Plasmodium* species, like *Plasmodium berghei* and *Plasmodium yoelii,* have been used as model species that can expedite studies of this pathogen. *P. yoelii* is an especially good model for investigating the mosquito and liver stages of development because key attributes closely resemble those of *P. falciparum*. Because of its importance, in 2002 the 17XNL strain of *P. yoelii* was the first rodent malaria parasite to be sequenced. Although this was a breakthrough effort, the assembly consisted of >5000 contiguous sequences that adversely impacted the annotated gene models. While other rodent malaria parasite genomes have been sequenced and annotated since then, including the related *P. yoelii* 17X strain, the 17XNL strain has not. As a result, genomic data for 17X has become the *de facto* reference genome for the 17XNL strain while leaving open questions surrounding possible differences between the 17XNL and 17X genomes. In this work, we present a high-quality genome assembly for *P. yoelii* 17XNL using PacBio DNA sequencing. In addition, we use Nanopore and Illumina RNA sequencing of mixed blood stages to create complete gene models that include coding sequences, alternate isoforms, and UTR designations. A comparison of the 17X and this new 17XNL assembly revealed biologically meaningful differences between the strains due to the presence of coding sequence variants. Taken together, our work provides a new genomic framework for studies with this commonly used rodent malaria model species.

Malaria remains a major global health burden (WHO Malaria Report 2022, (1)), with most of the 600,000 fatalities resulting from infection by human-infectious *Plasmodium falciparum*. The use of rodent-infectious model species has been instrumental to better understand those species that cause human disease due to high levels of genetic and physiological conservation across species (2). Researchers have routinely used these rodent model species, such as *Plasmodium yoelii*, *Plasmodium berghei*, and *Plasmodium chabaudi*, to investigate the entire *Plasmodium* life cycle, as genetic manipulations have long been rapid and rigorous in these species (2). We and others study *P. yoelii*, which is an especially good model for the mosquito and liver stages of *P. falciparum* parasite development (2). This is partly because *P. yoelii* mosquito stage parasites develop at a similar pace as do those of *P. falciparum* and their sporozoites are less promiscuous than *P. berghei* sporozoites (2). Because of this, many studies of genetically attenuated parasite vaccine candidates based upon sporozoites have recently included the use of *P. yoelii* as a preclinical model system (3). In support of this, large-scale analyses of gene expression of *P. yoelii* now match those available for *P. berghei* in many ways (4, 5, 6, 7, 8, 9). For these reasons, *P. yoelii* has been an important malaria parasite used as a proxy for *P. falciparum* in preclinical and discovery phase studies.

Intuitively, genetic studies of any species are best conducted with accurate genome assemblies and gene models. Therefore, several species of *Plasmodium* parasites were the subject of early whole-genome sequencing efforts in the late 1990s and early 2000s (10, 11). This work provided a genome assembly of the human-infectious *P. falciparum* parasite with 14 nuclear chromosomes and the 2 organellar genomes of its mitochondrion and apicoplast (11). In addition, gene models for *P. falciparum* were annotated with introns/exons, with further improvements establishing 5’/3′ untranslated regions (UTRs) and transcript isoforms (12, 13). Similarly, the rodent-infectious *P. berghei* ANKA parasite was originally sequenced in 2005, resulting in a genome assembly with 7497 contiguous sequences (contigs) that were later reduced to 16 contigs with a hybrid Illumina and 454 sequencing approach in 2014, and then further refined using PacBio sequencing in 2016 (14, 15, 16). Prior to this, the nonlethal *P. yoelii* 17XNL strain was the first rodent malaria parasite sequenced in 2002, which used ABI3700 sequencers and yielded a genome assembly of over 5000 contigs (10). The *P. yoelii* 17X strain, from which 17XNL was derived, was sequenced in 2014 alongside PbANKA using the same Illumina and 454 sequencing approach to similarly establish a 16 contig genome assembly (15). With the advent of more accurate long-read sequencing technologies, there has been a renewed interest in sequencing the *Plasmodium* genomes and transcriptomes, including those of another *P. yoelii* strain, PyN67, which has been used to study genetic polymorphisms and drug responses (17). In addition, the genomes of other apicomplexan parasites, such as *Cryptosporidium* and *Babesia* species, have now been established using a combination of long-read Nanopore sequencing and short-read Illumina sequencing (18, 19, 20).

Although their genomes have been updated and are conveniently provided on PlasmoDB.org, Py17X and PbANKA have gene models that largely reflect the coding sequences but not their UTRs despite the availability of RNA-seq data that could be used to approximate them (4, 5, 6, 7, 8, 9, 15, 21, 22, 23, 24, 25). Finally, while the 17XNL strain of *P. yoelii* remains a highly used laboratory strain worldwide, its reference genome and gene models have not been revisited since 2002, and thus its genome assembly and gene models remain highly fragmented and incomplete. As a result, most researchers use the genome assembly and gene models of the related *P. yoelii* 17X strain as a proxy when working with the 17XNL strain and must operate under the assumption that the genomes of the two strains are effectively the same. However, this prompts a few important questions. How similar are the 17X and 17XNL strains? In what ways are they truly suitable proxies for one another? While both 17X and 17XNL nonlethal strains can both spontaneously revert to a lethal phenotype, historically this has been more commonly reported for 17X and was an impetus for creating the 17XNL Clone 1.1 at NIH (26, 27). This history has also led to the use of 17XNL more predominantly in the United States, whereas 17X remains favored in Europe and other regions. Given the state of the 17XNL genome assembly and the limited gene models available for both strains, these practical questions surrounding the two strains could not be accurately addressed. However, these kinds of questions can now be more rigorously addressed with the inclusion of long-read DNA sequencing. The long sequence reads produced by PacBio and Nanopore approaches better facilitate the scaffolding of long, contiguous sequences in a *de novo* assembly, even for complex genomes that have extreme AT content and/or high degrees of repetitiveness, such as found with *Plasmodium* species (28, 29). In addition, by combining long-read and short-read sequencing, the strengths of each can be used to polish the assembly to reduce systematic errors introduced by each of the different methodologies.

Therefore, here we have created a high-quality reference genome and gene model annotation for the *P. yoelii* 17XNL strain that we have used to address these outstanding questions. We utilized HiFi PacBio DNA-seq to create a Py17XNL reference genome with 16 high-confidence/high-accuracy contigs. Even without any polishing efforts, this approach outperformed a parallel effort using a hybrid Nanopore long-read DNA-seq/Illumina short-read DNA-seq method by several key metrics, including its assembly quality and the reduction of gaps. Furthermore, we created gene annotations for genes transcribed in asexual and sexual blood stages using a combination of Nanopore direct RNA-seq and our preexisting Illumina RNA-seq datasets. These annotations include definitions of introns, exons, 5′, and 3′ UTRs, and transcript isoforms expressed in asexual and sexual blood stages. Using these data, we compared the genomic variance between the Py17XNL and Py17X strains to gain insight into the differences between the two strains and identified that most sequence variants reside in intergenic regions, while variation in the coding sequence of a select few genes could result in meaningful changes in Py17XNL parasite biology.

## Results

### A comparison of genome assembly approaches: PacBio HiFi *versus* Nanopore/Illumina hybrid sequencing

*P. yoelii* 17XNL remains a commonly studied rodent malaria strain. Yet, its genome assembly remains highly fragmented and consists of over 5000 contigs as generated in 2002 (10). Consequently, most researchers use the reference genome of the related Py17X strain as a substitute for Py17XNL without knowing how appropriate it is to use it as a genomic proxy (15). To resolve these questions, we created a high-quality genome assembly of *P. yoelii* 17XNL Clone 1.1 obtained from BEI Resources, which is the common origin of this strain of parasites for many laboratories. We explored several data analysis protocols for combining data from different sequencing platforms (*e.g.*, Illumina, Nanopore) to optimize this genome assembly. As widely available Nanopore sequencing chemistries (Q10) yield a systematic error, Nanopore data are often paired with Illumina data to provide error correction. The sequencing error rates from Illumina are typically above 99.9% and can be used with polishing algorithms to identify errors in assemblies that were produced with long, noisy reads (18, 19). We also generated high-fidelity PacBio sequencing data to access the feasibility of genome assembly without the need for error correction. A detailed outline of our experimental methods is included in Fig. S1.

To generate genomic DNA for this study, Swiss Webster outbred mice were infected with Py17XNL Clone 1.1 parasites that had been passaged only once following receipt from BEI Resources to create a genome assembly reflective of the current stocks available in the depository. High-molecular-weight genomic DNA was produced using the NEB Monarch HMW DNA Extraction Kit for Cells and Blood as described (30). DNA purity, quantity, and fragment lengths were determined to all be high quality by NanoDrop, Qubit, and TapeStation measurements, respectively (Table S1 and Fig. S2). Matched gDNA samples were sequenced on a Nanopore MinION and on an Illumina NextSeq 550. In parallel, Py17XNL gDNA was sequenced on a PacBio Sequel, and these raw reads were converted into circular consensus sequences using the Cirular Consensus Sequence algorithm (31).

**Figure 1:**
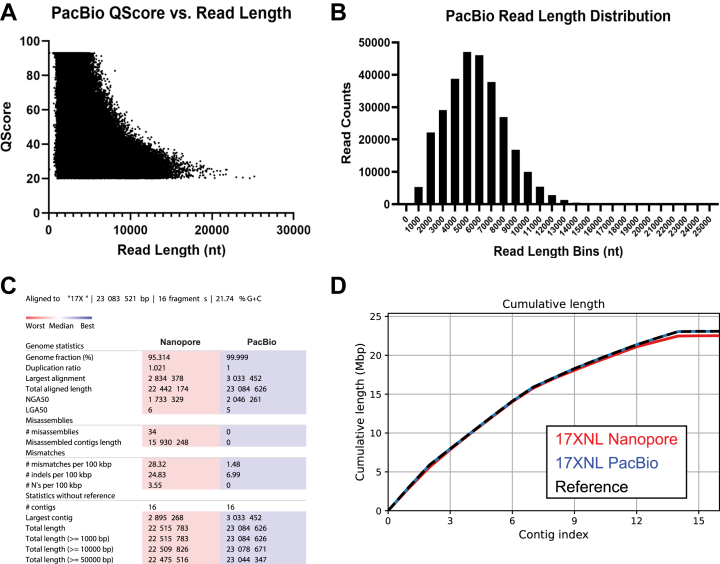
**PacBio HiFi high-quality long reads improve upon the preexisting Py17XNL genome and outperform a hybrid assembly approach with Nanopore and Illumina sequencing.***A*, QScore *versus* read length distribution for a PacBio sequencing run that was used to construct the final Py17XNL_2 genome assembly is presented. Note: HiFi PacBio sequencing has a minimum QScore threshold of 20 and a maximum QScore threshold of 93. *B*, a histogram is plotted to illustrate the distribution of PacBio read lengths. *C*, a comparison of assembly statistics between Nanopore and PacBio sequencing runs is provided. All statistics are based on contigs of size ≥ 500 bp. *D*, the cumulative length of contigs is plotted from largest to smallest.

To assess the quality of both Nanopore sequencing runs, we utilized Nanoplot, a quality control plotting suite specifically for long-read sequencing data (Fig. S3 and Table S2) (32). The Nanopore sequencing runs for replicates one and two resulted in an overall average read length of 16,706 bases, with an average Qscore of 11.3 (Table S2). We tested the high-accuracy basecalling algorithm that was released during this study and found it increased quality from 11.3 to 14.1 (Fig. S3 and Table S2). Because of this improvement, we used the high-accuracy basecalling algorithm for assessing our final datasets. Compared with the Nanopore data, the overall quality of the PacBio ccs reads were substantially higher, with an average Qscore of 36.3 (Fig. 1*A*). The biggest difference came with throughput, which increased to 1,660,222,360 bases with PacBio ccs reads as compared with 707,945,539 bases for Nanopore, while still maintaining an average read length of 5712.7 bases (Fig. 1*B* and Table S2).

**Figure 2:**
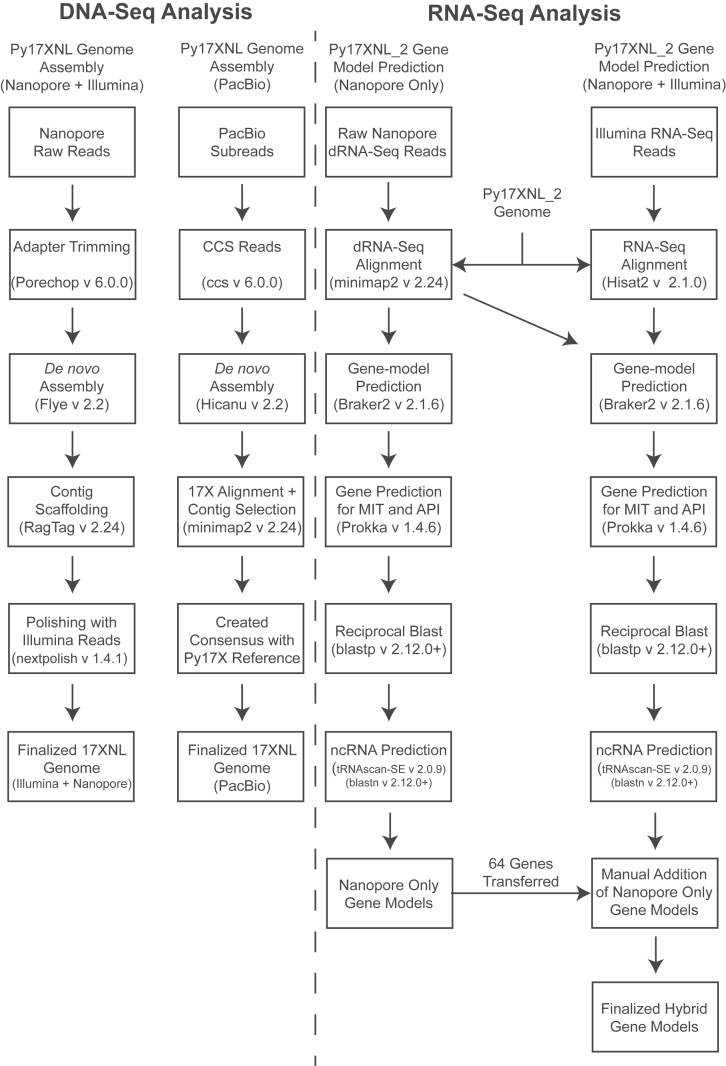
**Bioinformatics workflow used for genome assembly and annotation.** (*Left*) Genome assembly: high-accuracy ccs reads that were generated from PacBio subreads and trimmed Nanopore reads were *de novo* assembled to create draft genomes. Contigs were selected, and chromosome names were assigned based on the *P. yoelii* 17X reference genome alignment. Further processing of the Nanopore + Illumina hybrid assembly involved implementing scaffolding and iterative polishing. (*Right*) Gene-model prediction: a Nanopore dRNA-seq-based gene model and a hybrid gene model combining both Nanopore dRNA-seq and Illumina RNA-seq data were generated using Braker2. The predicted genes were annotated using reciprocal BLAST against *P. yoelii* 17X proteins. Illumina RNA-seq reads were previously reported (41).

We generated genome assemblies from both long-read datasets with the bioinformatic workflows described in Figure 2. To create the Nanopore/Illumina hybrid genome assembly, we assembled the Py17XNL Nanopore data using Flye to generate 26 contigs (Table S3) (33). The contigs were scaffolded using the Py17X genome as a guide in conjunction with the RagTag scaffolding program (33, 34). Finally, we layered error correction onto it in a multistep approach, first using nextpolish, followed by multiple rounds of consensus generation based on Illumina data alignment and variant calling (Fig. 2) (35). Through this process, we were able to reduce the number of contigs down to the expected 16, with a final genome coverage of 95% and the introduction of 34 misassembles as defined by the assembly evaluator program Quast (Fig. 1*C*) (36). The PacBio-based genome assembly was generated with the HiCanu program (37), which produced a *de novo* genome assembly with 132 contigs (Fig. 2). The resulting contigs were filtered to contain only the target species by aligning them against the Py17X genome using minimap2 (38). Contigs with a primary alignment length of less than 2% were denoted as contaminants and were removed, with many of these derived from the mouse host. The remaining contigs all matched the 17X reference chromosomes previously assembled by PacBio sequencing and were assigned their respective chromosome names (GCA_900002385.2). We observed that ∼1% of the subtelomeric/telomeric sequences were missing from our assembly due to the presence of heterogeneous repeated elements. Despite sufficiently high read depth and the use of highly accurate, long-read PacBio sequencing, these reads could not resolve the genome to a single haplotype. It is notable that similar observations have recently been made in a preprint describing the genome of the human-infectious *P. falciparum* parasite, which appears to harbor extrachromosomal plasmids bearing these subtelomeric sequences (39). A consensus genome was generated over these regions where we selected the haplotype reported for the 17X reference genome to enable cross-strain comparisons (File S1). Our final assembly consisted of a 23.08-Mb genome with 16 contigs (Fig. 1*C* and *D*, Table 1; Table S3). We have adopted the higher-quality PacBio-based genome assembly for the *P. yoelii* 17XNL strain for the rest of our analyses and for provision to the community on PlasmoDB.org, which we term Py17XNL_2 to distinguish it from the original genome assembly (Py17XNL_1) (10, 40). However, as both the hybrid Nanopore/Illumina and PacBio assemblies are potentially valuable to our research community, both assemblies have been publicly deposited in NCBI.

**Table 1:** Summary of finalized genome assembly and gene model creation statistics

Summary Statistics	17X (Otto *et al.* 2014)	17XNL_1 (Carlton *et al.* 2002)	17XNL_2 (This study)
Quast summary			
Number of contigs	16	5617 (5687^a^)	16
Assembled genome size	23.08 Mb	23.1 Mb	23.08 Mb
Genome fraction (%)	-	88.68%^b^	99.90%
Largest alignment	-	51.5 Kb	3,033,452
NGA50	-	7668^b^	2,046,261
LGA50	-	851^b^	5
Mismatches per 100 kb	-	72.72^b^	1.48
Indels per 100 kb	-	44.07^b^	6.99
N's per 100 kb	-	50^b^	0
BUSCO summary			
BUSCO genome completeness	98%^c^	83.9%^c^	97.60%
BUSCO protein completeness	99%^c^	64.7%^c^	90.00%
Annotation summary			
Number of genes	6263	7774 (5878^a^)	6077
Number of mRNAs	6041	7724	7047
Number of tRNAs	79	50 (39^a^)	66
Number of rRNAs	40	0 (7^a^)	40
Variant summary			
Variants with respect to 17X	-	N/D	1955
Number of substitutions	-	N/D	360
Number of insertions	-	N/D	833
Number of deletions	-	N/D	762
Number of variants in CDS	-	N/D	330

N/D, not determined.

a As reported in Carlton *et al.* Nature 2002.

b Determined using the Quast program for this study.

c Determined using a BUSCO analysis for this study.

### Nanopore direct RNA sequencing provides new information to preexisting gene models

We also set out to create more comprehensive gene models to increase the utility of the new Py17XNL_2 genome assembly for the *P. yoelii* 17XNL strain. In the currently available gene models for *P. berghei* (ANKA) and *P. yoelii* (17X, 17XNL) on PlasmoDB, only the coding sequences of genes are provided with no designation of UTRs, and little information is provided about alternatively spliced transcripts. We generated gene models that provide complete transcript information, including start and stop codons, transcription start and stop sites, and UTRs. Experimentally, we performed Nanopore direct RNA-seq in biological duplicate to generate long sequencing reads of asexual and sexual blood stage transcripts. RNA samples passed quality thresholds through analyses with Nanodrop, Qubit, and Bioanalyzer (Figs. S1 and S4; Table S1). These direct RNA-sequencing reads were quality controlled using Nanoplot with the same parameters described for Nanopore DNA-sequencing reads for both “Fast” and “High Accuracy” basecalling approaches (Fig. S5 and Table S2) (32). Similar to what was seen with our DNA samples, the high-accuracy basecalling algorithm provided the highest-quality data and was used for all final analyses in this study.

**Figure 3:**
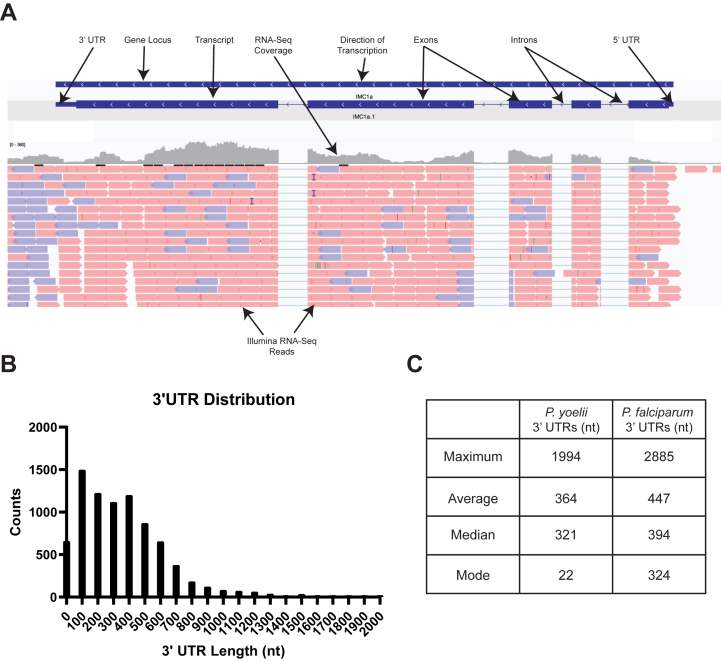
**Expanded *Plasmodium yoelii* 17XNL gene models leveraging RNA-seq data.***A*, an example gene model depicting IMC1a and its respective sequence features is provided. *B*, the 3′ UTR length distribution of all detected mRNAs is plotted as a histogram for chromosomal and mitochondrial genes. Transcripts encoded by the apicoplast are not polyadenylated and were not detected by Nanopore dRNA-seq. *C*, the maximum, average, median, and mode of the 3′ UTR lengths from all chromosomal and mitochondrial transcripts are compared with those from a *Plasmodium falciparum* dataset (13).

Gene models were created with two alternative methodologies using Nanopore direct RNA-seq long reads alone or in combination with our previously published Illumina short-read RNA-seq of mixed asexual and sexual blood stage parasites (Fig. 2) (41). These parallel approaches are both informative, given the strengths and limitations of both sequencing techniques. Nanopore direct RNA sequencing provides information that allows us to identify long/full-length sequencing reads that initiate at the 3′ end of mRNAs (42). However, when the full-length mRNA is not sequenced, less information is provided for the 5′ end (42). This limitation is remedied by the strong depth and breadth of sequencing coverage provided *via* Illumina sequencing. For both approaches, Nanopore RNA-seq reads were aligned to our Py17XNL_2 genome using minimap2 (38). For gene models created with both Nanopore and Illumina RNA-seq data, in parallel, the Illumina short reads were aligned to the Py17XNL_2 genome using Hisat2 (43). The exon–intron junction information from RNA-seq alignments provided additional support to the Braker2 gene model predictions (44). The Nanopore-only approach helped us identify 5683 genes, 5828 mRNAs, 66 tRNAs, and 40 rRNAs. Using the Nanopore/Illumina hybrid approach, we found 6077 genes, 7047 mRNAs, 66 tRNAs, and 40 rRNAs (Table 1). Of these genes, 866 were identified as expressing alternative transcript isoforms (Table S4). Gene models that were generated using both Nanopore and Illumina reads more closely matched the anticipated UTR length that was defined in recent *Plasmodium falciparum* transcriptomics data (13). A representative example of this more comprehensive gene model is illustrated in Figure 3*A*. In this study, we used the hybrid Nanopore and Illumina model for all downstream analyses, but we included both sets of gene models in the Supplemental material (File S2).

Nanopore direct RNA-seq initiates at the 3′ end due to the use of poly(dT) sequencing primers. As a result, significantly higher coverage was obtained for the 3′ UTRs than for 5′ UTRs for both sets of gene models. The higher coverage allows us to further analyze the 3′ UTR length distribution for *P. yoelii* 17XNL, which is of interest as *cis-*regulatory elements are often found in this portion of eukaryotic mRNAs (45). The transcripts typically have a UTR length between 100 and 200 nt, with a mean length of 364 nt. The largest UTR was reported for the H2B.Z histone variant mRNA, with 1994 nt (Fig. 3*B* and Table S5). Compared with the most up-to-date *P. falciparum* transcriptome, which used DAFT-Seq to resolve UTRs, *P. yoelii* 17XNL’s 3′ UTRs appear slightly shorter on average (Fig. 3*C*) (13).

### Comparisons between Py17XNL_1 and Py17XNL_2 demonstrates a greater degree of completeness for the new genome assembly

**Figure 4:**
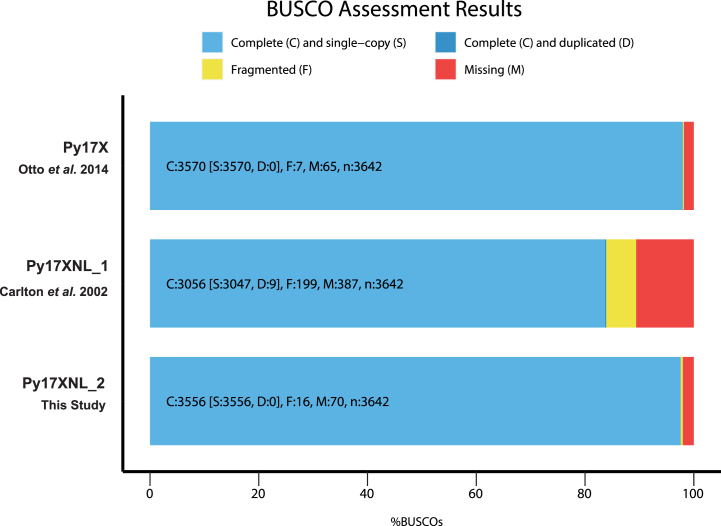
**BUSCO analysis demonstrates genome assembly completeness.** Of the 3642 BUSCO groups that were searched, 3556 single-copy BUSCOs were found to be present in the 17XNL_2 assembly resulting in a completeness score of 97.6%. The BUSCO results for Py17XNL_1 (83.9%) and Py17X (98.0%) reference genomes are also shown for comparison.

Using the Py17XNL_2 genome assembly and associated gene models, we compared our results with the original Py17XNL genome (Py17XNL_1) and the Py17X reference genome. As anticipated, there was a substantial reduction in the number of gaps/misassembles and greater genome coverage when comparing Py17XNL_2 *versus* Py17XNL_1 (Table 1). Although our Nanopore/Illumina-based genome assembly (Py17XNL Nanopore) contained the same number of contigs as the PacBio-based Py17XNL_2 assembly, in our assessment, the PacBio-based assembly provides a more accurate reference genome for future research uses. The 6077 new gene annotations more accurately represent the anticipated number of genes for Py17XNL and more closely match those annotated in other *Plasmodium* species (Table 1). Moreover, these new gene models include coding sequences, UTRs, and transcript isoforms, which are lacking in the provided gene models currently available on PlasmoDB for *P. yoelii* and *P. berghei* parasite (Tables S4 and S5). In addition to these assessment metrics, we determined the completeness of the reference genome based on marker genes. To quantify this, we used a Benchmarking Universal Single-Copy Orthologs (BUSCO) analysis that detects whether a predefined set of single-copy marker genes in the *Plasmodium* lineage is present in these data (Fig. 4) (46). This BUSCO dataset contains 3642 BUSCO groups from 23 different species, including *P. falciparum* 3D7, *P. yoelii* 17XNL, *Plasmodium vivax, P. berghei* ANKA, *P. chabaudi,* and others. From this search, 3556/3642 (97.6%) of complete and single copy BUSCOs were found to be present, indicating that this genome assembly and gene annotation has a high level of completeness (Fig. 4).

### Variation between the Py17XNL and Py17X reference genomes primarily resides in the intergenic regions and the ends of chromosomes

**Figure 5:**
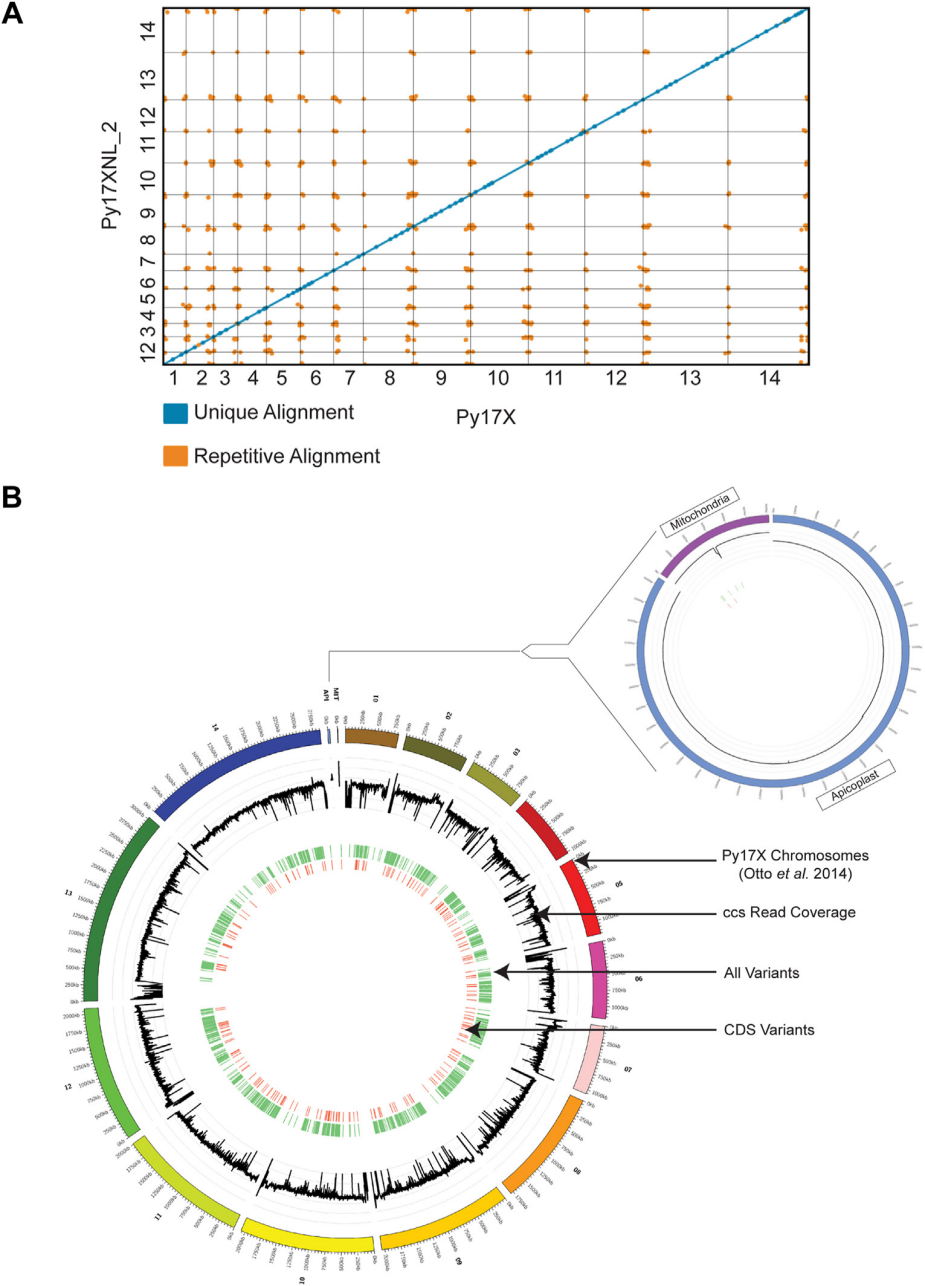
**Differences between the *P. yoelii* 17X and 17XNL_2 assemblies.***A*, the Py17XNL_2 reference genome was mapped to Py17X to determine their degree of similarity. A dot plot depicting this agreement is shown, with blue lines denoting unique alignments and orange lines depicting repeat regions. *B*, a circos plot is presented with the following tracks listed from outside to inside: (1) Py17X reference genome, (2) Py17XNL_2 ccs read coverage in the natural log scale (minimum value of 0 and maximum value of 8 with the lower bound closer to the center), (3) SNPs and indels between the two genomes are shown in *light green*, (4) SNPs and indels in the coding sequence of genes are shown in *orange*. An expanded view that includes the apicoplast and mitochondria is shown separately. A short deletion in the mitochondrial genome compared with Py17X is evident.

As Py17X is commonly used as an interchangeable proxy genome for Py17XNL, we sought to determine what similarities and differences exist between the strains and how the differences may impact genetic studies. We performed chromosome-wide alignments between the Py17X and Py17XNL_2 genomic builds using the minimap2 (38) program and assessed the genome-wide variants with paftools. We observed extensive linear agreement between the two strains, with 99.9% of the Py17X genome matching with the Py17XNL_2 genomic build (Fig. 5*A*). We did not detect any large structural variation between the strains, a finding also supported through our alternative Py17XNL_2 Nanopore/Illumina genome build. At the same time, we also identified a total of 1955 potential single nucleotide/short variants across the two strains, the majority (62%) of which were found in intergenic regions (Fig. 5*B*). We found that the apicoplast genome was identical between strains, whereas the Py17XNL_2 mitochondrial genome has a 127-bp deletion in the middle of its sequence (see Fig. 5*B*, Inset). Compared with Py17X, the deletion is located in the intergenic region between *cox1* (PY17X_MIT00800) and a ribosomal RNA fragment annotated as PY17X_MIT00700. Together, we conclude that, while these two strains are highly similar, there are sequence differences that may be functionally relevant.

**Figure 6:**
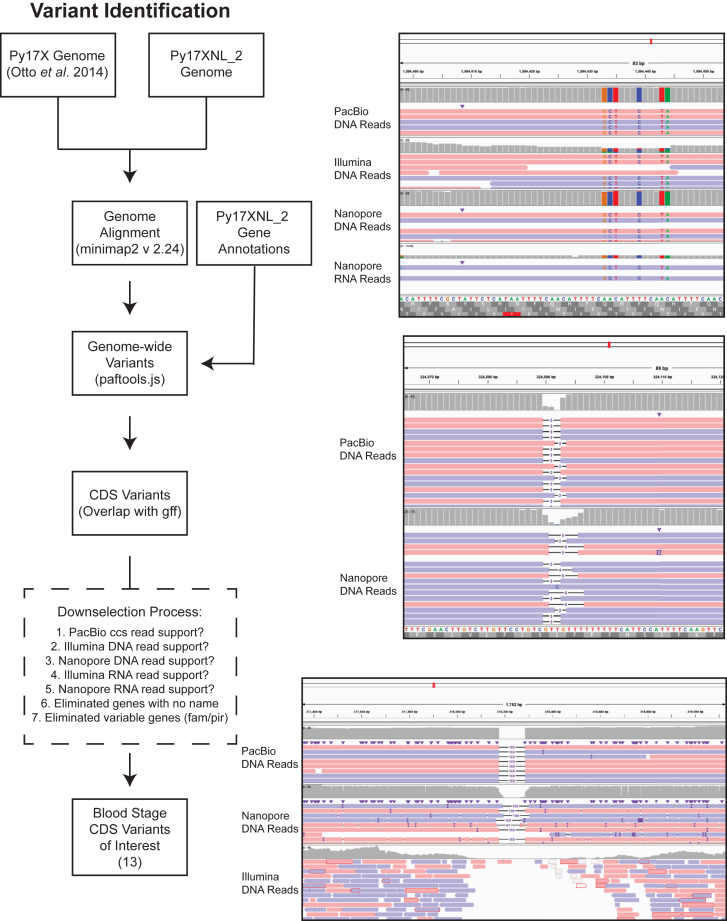
**Identification of blood stage–expressed variants between 17X and 17XNL_2.** (*Left*) Variants of interest that are expressed in blood-stage parasites were chosen based on the presence of the variant sequence within the coding sequence, the extent to which the variant calls are supported by sequencing data, and if the gene has been named. Downselected genes are further described in Table S6. To be considered, at least two sequencing methods are needed to support the variant call, with at least 80% of the reads in agreement and a minimum of five reads at the position (two read minimum for Nanopore). (*Right*) IGV snapshots with representative examples of different variants found in AP2-SP (PY17XNL_1303202), RAD50 (PY17XNL_0104722), or CSP (PY17XNL_0404050) are presented *top to bottom*.

**Figure 7:**
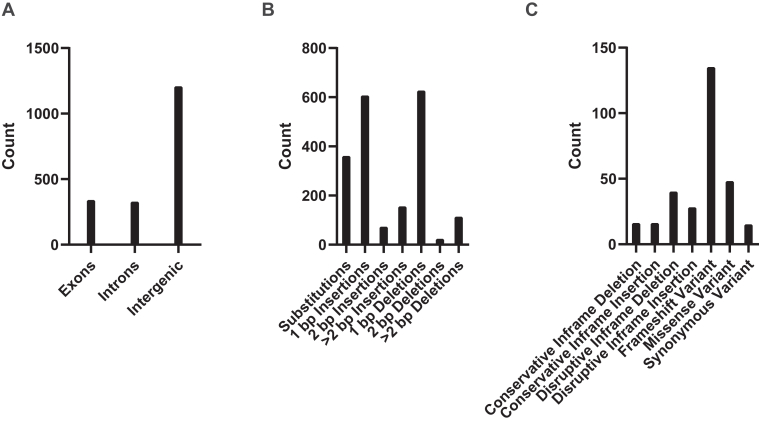
**The location and potential impact on translation of variants between 17X and 17XNL_2 genome assemblies.***A*, the distribution of variant locations throughout the entire Py17XNL_2 genome is shown. *B*, the types of variants represented within the Py17XNL_2 genome with their respective counts are plotted. *C*, the distribution of variant types within coding sequences is depicted as a bar graph.

To further determine the potential impacts of these genome variants, we characterized the position of variants with respect to nearby genes and, when applicable, determined the specific DNA and amino acid changes that would result from the change. Most variants were found to be located in intergenic regions and were characterized as single base pair indels (Figs. 6 and 7, *A* and *B*). In addition, 330 variants fell within coding regions, which we also manually curated and verified using sequencing data from multiple platforms used in this study. For this, we determined if the variant had sufficient PacBio ccs read support (>80% of reads supporting the variant, with at least 5x coverage at the region), and when possible, we also determined if additional Nanopore or Illumina DNA- or RNA-sequencing reads supported the variant (>80% of reads supporting the variant with at least 5x coverage for Illumina sequencing and 2x coverage for Nanopore sequencing). Owing to the overrepresentation of single-base-pair indels, the majority of amino acid changes lead to frameshifts (Fig. 7*C*). Most of these frameshifts were observed in genes annotated as “hypothetical proteins” that have no known function or are members of multigene families like *pir* or *fam* genes, requiring further investigation to determine the biological impacts of these differences.

Through manual curation, a substantial number of variants had support from at least three sequencing platforms. Owing to the strict thresholds of this variant calling process, some sequencing methods did not capture the variant sufficiently enough to provide support, typically due to a lack of coverage at the position of the variant. An example of this occurring is with CSP, which had a large deletion that was adequately supported by PacBio and Nanopore DNA-seq data (Fig. 6). Illumina DNA-seq reads instead have a complete loss of coverage, with only one read correctly mapping to this repetitive region (Fig. 6, bottom panel). We believe that this observation is an example of the advantages of long-read sequencing platforms that can identify variants that may be missed when using short-read sequencing alone.

Based upon these criteria, we also separated genes that were expressed in asexual/sexual blood stages due to sequencing support from either Illumina or Nanopore direct RNA-seq and that were further validated through manual curation and created different variant lists accordingly (Table S6). Finally, we filtered out genes with no annotated gene name and those that belong to a variable gene family (fam/pir gene families) (Fig. 6). After this filtering, we focused our analyses on 13 blood stage–expressed genes in which variations have a high potential to affect biological functions (Table 2). Although the biological implications of the differences between Py17X and Py17XNL will need further experimental validation, many variants could have profound impacts. One such example is *ap2-sp*, which has both synonymous and missense variants between the AT-hook and AP2 domain (47, 48). AP2-SP is an ApiAP2 transcription factor with many target genes that are expressed specifically in the sporozoite stage of the *Plasmodium* life cycle (47, 49, 50). It has also been shown that disruption of this gene results in the loss of sporozoite formation entirely in the related *P. berghei* parasite and has important activities in blood stages in *P. falciparum* (47, 49, 50, 51). Another affected gene is *pk4*, which encodes an essential eIF2α kinase–related enzyme and contains changes in its noncytoplasmic domain as determined by InterPro domain predictions across 17X and 17XNL strains (52, 53, 54, 55). Further study of these genes and several other candidates is warranted to understand the biological role these variants may play across strains.

**Table 2:** Prioritized list of coding sequence variants between the Py17X and Py17XNL genomes

Py17X gene ID	Py17XNL gene ID	Gene name	Chromosome position	Py17X DNA	Py17XNL DNA	Mutation type	AA change	Protein alignment
PY17X_0811400	Py17XNL_0801678	Proteasome subunit alpha type-3.1	529296	G	A	Missense variant	Val24Ile	Py17X MAGLSAGYDLSVSTFSPDGRLYQVEYIYKAINNNNTSISLECKDGVISCSINTSLEKNKMIKKNSYNRIYYV
								17XNL MAGLSAGYDLSVSTFSPDGRLYQIEYIYKAINNNNTSISLECKDGVISCSINTSLEKNKMIKKNSYNRIYYV
								Cons ∗∗∗∗∗∗∗∗∗∗∗∗∗∗∗∗∗∗∗∗∗∗∗:∗∗∗∗∗∗∗∗∗∗∗∗∗∗∗∗∗∗∗∗∗∗∗∗∗∗∗∗∗∗∗∗∗∗∗∗∗∗∗∗∗∗∗∗∗∗∗∗
PY17X_0316900	Py17XNL_0303734	Plasmodium exported protein	625346	G	A	Missense variant	Gly1324Ser	Py17X PEQKENGDIGEASNNAAELKENMNDLLKDTIEISKESIKEHDAQSIMFTRKFIKHVSGYDIQKAKDHPTDED
								17XNL PEQKENGDIGEASNNAAELKENMNDLLKDTIEISKESIKEHDAQSIMFTRKFIKHVSSYDIQKAKDHPTDED
								Cons ∗∗∗∗∗∗∗∗∗∗∗∗∗∗∗∗∗∗∗∗∗∗∗∗∗∗∗∗∗∗∗∗∗∗∗∗∗∗∗∗∗∗∗∗∗∗∗∗∗∗∗∗∗∗∗∗∗.∗∗∗∗∗∗∗∗∗∗∗∗∗∗
PY17X_1022000	Py17XNL_1002268	PP7	964836	C	A	Missense variant	Leu342Phe	Py17X FAFKLSNYDSVIINRGNHECSYMNEVYGFHNEVLSKYDESVFDIFQEIFELLSLSVNIQNQIFVVHGGLSRY
								17XNL FAFKLSNYDSVIINRGNHECSYMNEVYGFHNEVLSKYDESVFDIFQEIFELLSFSVNIQNQIFVVHGGLSRY
								Cons ∗∗∗∗∗∗∗∗∗∗∗∗∗∗∗∗∗∗∗∗∗∗∗∗∗∗∗∗∗∗∗∗∗∗∗∗∗∗∗∗∗∗∗∗∗∗∗∗∗∗∗∗∗:∗∗∗∗∗∗∗∗∗∗∗∗∗∗∗∗∗∗
PY17X_1419800	Py17XNL_1401046	ACDC domain-containing protein	839701	G	T	Missense variant	Arg1562Ile	Py17X VNENFTAELNGVQMYNGNEKKKKKKNYSLSINKNNGNIKDNENTNEILLRYENEVYAPNNDVEKNLIEDNNI
								17XNL VNENFTAELNGVQMYNGNEKKKKKKNYSLSINKNNGNIKDNENTNEILLIYENEVYAPNNDVEKNLIEDNNI
								Cons ∗∗∗∗∗∗∗∗∗∗∗∗∗∗∗∗∗∗∗∗∗∗∗∗∗∗∗∗∗∗∗∗∗∗∗∗∗∗∗∗∗∗∗∗∗∗∗∗∗:∗∗∗∗∗∗∗∗∗∗∗∗∗∗∗∗∗∗∗∗∗∗
PY17X_0106400	Py17XNL_0104725	RNA-binding protein	339741	AGATAGG	A	Disruptive in-frame deletion	Asp578_Arg579del	Py17X RDRDRDRDRDRDRDRDRDRDRDRR
								17XNL RDRDR--DRDRDRDRDRDRDRDRR
								Cons ∗∗∗∗∗ ∗∗∗∗∗∗∗∗∗∗∗∗∗∗∗∗∗
PY17X_1206500	Py17XNL_1204935	UTP25	344114	GGAAAATGGGAAT	G	Disruptive in-frame deletion	Gly163_Asn166del	Py17X ENGNENGNENGNENGNENGNENDKNGNDKNGNDKNGNDKNEASSFQSKDEIYMNILINNIKSQNEDFLNVKE
								17XNL ENGNENGNENGNENGNEN----DKNGNDKNGNDKNGNDKNEASSFQSKDEIYMNILINNIKSQNEDFLNVKE
								Cons ∗∗∗∗∗∗∗∗∗∗∗∗∗∗∗∗∗∗ ∗∗∗∗∗∗∗∗∗∗∗∗∗∗∗∗∗∗∗∗∗∗∗∗∗∗∗∗∗∗∗∗∗∗∗∗∗∗∗∗∗∗∗∗∗∗∗∗∗∗
PY17X_1110800	Py17XNL_1105517	KH domain-containing protein	539935	G	GTTA	Disruptive in-frame insertion	Asn1809dup	Py17X NNNVGRDNIIRKENKGIMMHDDKDKFSKGGNNRYFGDKTNNFNNKN-NNNNNNNNNNNNNNNNNNAKNNYLSKDSMI
								17XNL NNNVGRDNIIRKENKGIMMHDDKDKFSKGGNNRYFGDKTNNFNNKNNNNNNNNNNNNNNNNNNNNAKNNYLSKDSMI
								Cons ∗∗∗∗∗∗∗∗∗∗∗∗∗∗∗∗∗∗∗∗∗∗∗∗∗∗∗∗∗∗∗∗∗∗∗∗∗∗∗∗∗∗∗∗∗∗ ∗∗∗∗∗∗∗∗∗∗∗∗∗∗∗∗∗∗∗∗∗∗∗∗∗∗∗∗∗∗
PY17X_1334500	Py17XNL_1303202	AP2-SP	1594433	AAC, T, AC	GCT, C, TA	Synonymous and missense variant	Val133Tyr, Val136Ser	Py17X QINYNISNDIMNTVPSTNCDVTHDSVSSVPNNAFENVENVKNVENVENVKNVENVENVENVENVENVENYEN
								17XNL QINYNISNDIMNTVPSTNCDVTHDSVSSVPNNAFENVENVKNVENVENVKNVENVENVENYENSENVENYEN
								Cons ∗∗∗∗∗∗∗∗∗∗∗∗∗∗∗∗∗∗∗∗∗∗∗∗∗∗∗∗∗∗∗∗∗∗∗∗∗∗∗∗∗∗∗∗∗∗∗∗∗∗∗∗∗∗∗∗∗∗∗∗ ∗∗ ∗∗∗∗∗∗∗∗
PY17X_1128400	Py17XNL_1105678	PK4	1187719	T	TGAA	Conservative in-frame insertion	Glu266dup	Py17X FYNSYNYCNNNNSKRDEKIEKNIVEKNIENKYNIKEYDKTNKSILFPIE-EFKKIIQIENNIERNYIVPKES
								17XNL FYNSYNYCNNNNSKRDEKIEKNIVEKNIENKYNIKEYDKTNKSILFPIEEEFKKIIQIENNIERNYIVPKES
								Cons ∗∗∗∗∗∗∗∗∗∗∗∗∗∗∗∗∗∗∗∗∗∗∗∗∗∗∗∗∗∗∗∗∗∗∗∗∗∗∗∗∗∗∗∗∗∗∗∗∗ ∗∗∗∗∗∗∗∗∗∗∗∗∗∗∗∗∗∗∗∗∗∗
PY17X_1451200	Py17XNL_1401341	BDP5	2018932	A	AAATATAAAC	Disruptive in-frame insertion	Asn320_Asn321insAsnIleAsn	Py17X NKIRSKNEINNSPNTDKVEKNIN---NINNINNINNNTNNNNVHEYVPNNLNDEFIEEKKLDKNKFNEYKNN
								17XNL NKIRSKNEINNSPNTDKVEKNINNINNINNINNINNNTNNNNVHEYVPNNLNDEFIEEKKLDKNKFNEYKNN
								Cons ∗∗∗∗∗∗∗∗∗∗∗∗∗∗∗∗∗∗∗∗∗∗∗ ∗∗∗∗∗∗∗∗∗∗∗∗∗∗∗∗∗∗∗∗∗∗∗∗∗∗∗∗∗∗∗∗∗∗∗∗∗∗∗∗∗∗∗∗∗∗
PY17X_0942100	Py17XNL_0900429	PAIP1	1662686	A	AAAT	Disruptive in-frame insertion	Asn1941dup	Py17X NVNKNKEIGKDEIQINSQINNLDDNAKGKKSNIFNQAKSSYKYPAEEGENNSNTNTSTEN-NNNNNNNNNNKT
								17XNL NVNKNKEIGKDEIQINSQINNLDDNAKGKKSNIFNQAKSSYKYPAEEGENNSNTNTSTENNNNNNNNNNNNKT
								Cons ∗∗∗∗∗∗∗∗∗∗∗∗∗∗∗∗∗∗∗∗∗∗∗∗∗∗∗∗∗∗∗∗∗∗∗∗∗∗∗∗∗∗∗∗∗∗∗∗∗∗∗∗∗∗∗∗∗∗∗∗ ∗∗∗∗∗∗∗∗∗∗∗∗
PY17X_0106100	Py17XNL_0104722	RAD50	324089	CGTT	C	Disruptive in-frame deletion	Gln834del	Py17X ENITNCVNKNEDILSDNLIKLESKKRVTAHFEELENGMKKKQRQEQDKFETVQKMKIEKIEKISKIEKINKI
								17XNL ENITNCVNKNEDILSDNLIKLESKKRVTAHFEELENGMKKK-RQEQDKFETVQKMKIEKIEKISKIEKINKI
								Cons ∗∗∗∗∗∗∗∗∗∗∗∗∗∗∗∗∗∗∗∗∗∗∗∗∗∗∗∗∗∗∗∗∗∗∗∗∗∗∗∗∗ ∗∗∗∗∗∗∗∗∗∗∗∗∗∗∗∗∗∗∗∗∗∗∗∗∗∗∗∗∗∗
PY17X_1001900	Py17XNL_1204888	Erythrocyte membrane antigen 1	174565	TAAATGA	T	Conservative in-frame deletion	Asn287_Glu288del	Py17X SYLNNGENAEDQELDDEVASCFADGENVNDKELDEVISYLANGENVNVNVNENVNENVNENVNENENENENE
								17XNL SYLNNGENAEDQELDDEVASCFADGENVNDKELDEVISYLANGENVNVNVNENVNENVNENVNE--NENENE
								Cons ∗∗∗∗∗∗∗∗∗∗∗∗∗∗∗∗∗∗∗∗∗∗∗∗∗∗∗∗∗∗∗∗∗∗∗∗∗∗∗∗∗∗∗∗∗∗∗∗∗∗∗∗∗∗∗∗∗∗∗∗∗∗∗∗ ∗∗∗∗∗∗

AA, amino acid.

Among the non-blood stage expressed genes, *trap, lisp2,* and *csp* all had variants in their coding sequences relative to 17X. Of these, the most notable one is a large in-frame deletion within the repeat region of CSP, leading to the loss of six of the repeating units of D/PQGPGA in Py17XNL (Fig. S6). This same gene in the YM strain of *P. yoelii* is even shorter and lacks an additional repeating unit compared with 17XNL (Fig. S6) (15). In *P. berghei*, it was found that 25% of this repeat region could be eliminated before impacting parasite development, which is approximately the length reduction observed in 17XNL and YM as compared with 17X (56). Therefore, our observation may reflect a minimum repeat length necessary for CSP functions.

## Discussion

Here we have created a high-quality genome assembly with experimentally validated gene models for the commonly used 17XNL strain of the *P. yoelii* malaria parasite species. We envision our assembly as a useful resource to the malaria research community, as it provides a much-needed update to the Py17XNL_1 reference genome, which was among the first to be completed in the early days of the genomics era 20 years ago (10). By directly comparing the strengths and genome assemblies created from either PacBio HiFi sequencing or a combination of Nanopore DNA-seq and Illumina DNA-seq, we identified that, while the hybrid Nanopore/Illumina approach yielded a robust genome assembly, the PacBio HiFi-based assembly consisted of fewer misassembles and covered a greater fraction of the genome. Therefore, we have chosen the PacBio-based genome assembly as our best new reference genome for the *P. yoelii* 17XNL strain, which we have designated as Py17XNL_2 within this study. Our findings align with many recent studies conducted to improve the reference information on *Plasmodium* species, which also utilized an exclusively PacBio-based approach (12, 14, 17, 57). We also deemed it important to conduct both approaches to leverage the strengths of Nanopore sequencing, which permit greater detection of large-scale structural variants in the genome as compared with approaches with shorter read lengths (28). The strengths of Nanopore sequencing have also been leveraged by other sequencing efforts, most notably the recent telomere-to-telomere sequencing effort of the human genome that used ultra-long read approaches (58). During this study, advances in Nanopore basecalling software were made that enabled more accurate sequencing without the need for resequencing or new hardware. We therefore directly compared the previous “fast” *versus* new “high-accuracy” basecalling algorithms and observed a substantial increase in Qscores associated with the same DNA and RNA sequencing data (Table S2). However, even with the use of high-accuracy basecalling, PacBio data still enabled the most accurate Py17XNL genome assembly and covered the greatest fraction of the genome. Of special note, we determined that our final genome assembly could not be resolved to a single, unambiguous haplotype due to the presence of sequence variation in the subtelomeric regions of some chromosomes. We observed that some regions had substantial and robust read support for more than one genomic sequence, which in some cases reflected only a small number of nucleotide differences. Moreover, we also confirmed this sequence variance through manual inspection of the data. As this phenomenon is likely present in genome assemblies for other species/strains, we recommend that researchers with a special interest in these subtelomeric regions manually inspect those data as needed. Finally, another explanation for the presence of robustly identified, highly related subtelomeric sequences may be provided by a recent preprint describing the presence of extrachromosomal plasmids harboring subtelomeric sequences in the human malaria parasite *P. falciparum* (39).

To provide an even more useful genome reference, here we also provide new gene annotations for the 17XNL strain of *P. yoelii* to facilitate more reliable forward and reverse genetic studies of this key model malaria species. Regardless of the rodent malaria RNA-seq studies that have been performed, gene models available on PlasmoDB for *P. berghei* and *P. yoelii* only consist of their putative coding sequences. In this study, we now report experimentally validated information on alternatively spliced transcripts and UTRs of Py17XNL blood stage–expressed genes. To date, the only other comparable efforts in our field have been applied to *P. falciparum* with a focus on either identifying alternatively spliced transcripts or experimentally defining and annotating long noncoding RNAs (lncRNAs) (59, 60, 61). In addition, because Nanopore direct RNA-seq reads initiate at the 3′ end of mRNAs and progress toward the 5′ end, the approach is well suited for providing information about the 3′ UTRs of a population of mRNAs. From our data, we have created both a Nanopore-only and a Nanopore/Illumina hybrid gene model annotation that can both be useful to researchers depending on the questions they are pursuing. These new gene models include well-defined 3′UTRs for Py17XNL that are in agreement with the length distribution of those described for *P. falciparum* (13). Owing to the strengths of this approach, we anticipate that Nanopore direct RNA sequencing will become a useful tool for future work on *Plasmodium* parasites, especially as sequencing chemistry and basecalling algorithms improve.

Using the greatly improved Py17XNL reference genome, we were able to critically analyze, manually curate, and verify genomic variation across the 17X and 17XNL strains of *P. yoelii*. As it is currently common practice to use Py17X as a proxy genome for Py17XNL for genomic studies, we thought it was important to begin addressing whether biologically relevant differences were present that would impact such efforts. By aligning the two genomes, we saw that there was an excellent linear agreement between them, with most variation taking place in intergenic regions. In total, there were 1955 variants across the entire sequence, with 330 of those being in the coding sequence of genes. Most of these variants were single-base-pair indels that most likely accounted for the overrepresentation of frameshift variants in the respective amino acid sequence. Upon further analysis of these variants, some interesting questions arose regarding the biological implications that these changes could have. Specific examples of genes with impactful variants include the ApiAP2 transcription factor AP2-SP and PK4, which are essential for *Plasmodium* development and warrant follow-up studies (47, 49, 50, 52, 54, 55). Aside from these blood stage–expressed genes, it is also important to note the large-scale differences between the 17X, 17XNL, and YM strains of *P. yoelii* in the central repeat of CSP, which are 150, 114, and 108 amino acids long, respectively (Fig. S6). The in-frame deletions result in truncations of entire six-amino-acid repeating units of D/PQGPGA, with 17XNL having six fewer units and YM having seven fewer than 17X. In *P. berghei*, it was found that 25% of this repeat region could be eliminated before impacting parasite development, which reflects the approximate reduction in repeat length in 17XNL and YM strains as compared with 17X (56). We anticipate that this may reflect a minimum repeat length that is applicable to both highly related species. This is indirectly corroborated by the absence of any reports that have documented significant differences in sporozoite development, functions, or transmissibility between the 17X and 17XNL strains. In addition, this particular variant was identified in both Nanopore and PacBio long-read DNA-sequencing datasets, with Illumina short-read sequencing lacking coverage at this site to accurately identify this deletion (Fig. 6).

This *P. yoelii* 17XNL_2 reference genome and its more comprehensive gene annotations provide a resource that we believe will be helpful to the rodent malaria research community. We stress that, while most genes are identical between the 17X and 17XNL strains, there are appreciable genomic differences in important genes that should be considered when conducting genomic studies. Therefore, we conclude that, while 17X may be a suitable genomic proxy for 17XNL in many cases, for genes where variance exists, such as *csp, trap, lisp2, ap2-sp, pk4*, and others, our assembly will provide a better reference dataset. In addition, our improved annotations with complete transcript information is better suited for RNA-seq studies that operate on transcript abundances values.

## Experimental procedures

### Animal experiments statement

All animal care strictly followed the Association for Assessment and Accreditation of Laboratory Animal Care (AAALAC) guidelines and was approved by the Pennsylvania State University Institutional Animal Care and Use Committee (IACUC# PRAMS201342678). All procedures involving vertebrate animals were conducted in strict accordance with the recommendations in the Guide for Care and Use of Laboratory Animals of the National Institutes of Health with approved Office for Laboratory Animal Welfare (OLAW) assurance.

### Experimental animals

Female Swiss Webster mice, 6 to 8 weeks old, from Envigo were used for all experiments in this work.

### Parasite preparation and isolation

Mice were infected with wildtype Py17XNL Clone 1.1 parasites from BEI Resources until a parasitemia between 1 to 3% was reached. Approximately 1 ml of blood was collected from each euthanized mouse, which was then added to 5 ml of heparinized (200 U) 1X PBS to prevent coagulation. The infected blood was spun, and the serum was aspirated to isolate the red blood cells. Cells were resuspended in 10 ml 1X PBS and then passed through a cellulose column (Sigma #C6288) to remove mouse leukocytes. The red blood cells were then lysed in 0.1% w/v saponin in 1X PBS for 5 min at room temperature, and parasite pellets were subsequently washed in 10 ml 1X PBS.

### gDNA preparation

All gDNA samples used for Nanopore, Illumina, and PacBio sequencing were prepared using the NEB Monarch HMW DNA Extraction Kit for Cells and Blood (NEB #T3050) using the manufacturer’s protocol for fresh blood with slight modifications as we have described (30). Briefly, the saponin-lysed parasite pellet was resuspended in 150 μl of Nuclei Prep Buffer containing RNase A. After resuspension of the pellet, 150 μl of Nuclei Lysis Buffer containing Proteinase K was added and mixed by inversion. The sample was then placed in a thermal mixer at 56 °C with an agitation speed of 1500 rpm for 10 min, which reflects the maximum agitation speed possible for this instrument. Next, 75 μl of precipitation enhancer was added and mixed by inversion. Two DNA capture beads were added to the tube, along with 275 μl of isopropanol. The sample was then mixed 30 times with manual, slow, end-over-end inversions (instead of an end-over-end rotator) to ensure the gDNA stuck to the capture beads. The supernatant was removed, and the beads were washed twice with 500 μl of gDNA wash buffer. Subsequently, 100 μl of elution buffer II was added and the sample was incubated for 5 min at 56 °C in a thermal mixer with agitation at 300 rpm. The beads were added to a bead retainer in an Eppendorf tube, and the sample was spun down for 30 s at 12,000*g*. All samples were stored at 4 °C to minimize shearing from freeze-thaw cycles. Fresh gDNA samples were made for replicate 1 and replicate 2 for Nanopore sequencing and the sole sample for PacBio. The same gDNA samples used for Nanopore replicates 1 and 2 were used for Illumina DNA sequencing replicates 1 and 2. Sample concentration and purity were assessed *via* Qubit and Nanodrop, respectively (Thermo Fisher Scientific Nanodrop 2000 and Qubit instruments with the Qubit dsDNA BR Assay Kit [Cat #Q32853]). Fragment length was assessed using an Agilent Technologies TapeStation 4200 system with Genomic DNA ScreenTapes (Cat #5067-5366 and 5067-5365).

### RNA preparation

RNA samples were prepared from two biological replicates for Nanopore direct RNA sequencing. RNA samples were produced using the Qiagen RNeasy kit using the manufacturer’s protocol with slight modifications to improve yield (Cat # 74104). Briefly, 350 μl of Buffer RLT was added to resuspend the parasite pellet. The sample was homogenized only by passage through a 20-gauge needle five times, and it was put back into the same microfuge tube. Next, 350 μl of 70% ethanol was added and mixed by pipetting using wide-bore pipettes. The sample was then added to the spin column and was centrifuged for 15 s at 8000*g*. The column was washed twice with 500 μl RPE buffer and was again centrifuged for 15 to 60 s at 8000*g*. Residual ethanol was removed by a final spin at these parameters. RNA was eluted from the column into a fresh microfuge tube with 30 μl of diethyl pyrocarbonate (DEPC)-treated water. The sample was incubated for 15 min at room temperature to improve recovery yield. The sample was then collected by centrifugation for 1 min at 8000*g*. A second elution with 30 μl DEPC-treated water was performed as above to improve yield. To eliminate contaminating DNA, an off-column Dnase I digestion was performed as per the Sigma #AMPD1 technical bulletin. Briefly, 8 μl of the prepared RNA was mixed with 1 μl 10X Reaction buffer and 1 μl Dnase I, Amplification Grade, 1 unit/μl (Cat # AMPD1-KT). The sample was gently mixed and incubated at room temperature for 30 min. The digestion was terminated by the addition of 1 μl stop solution, followed by heat inactivation of the Dnase I. RNA was precipitated with ethanol by adding 0.1 volume of 3 M sodium acetate pH 5.5 at room temperature (RT), 4 volumes of reagent grade 200 proof ethanol, and 0.5 μl 20 mg/ml glycogen. The solution was allowed to precipitate overnight at −80 °C. The solution was then spun down at 4 °C at 12,000*g* for 10 min. The supernatant was aspirated, and 1 ml of 70% ethanol was added to wash the pellet. The pellet was spun down as above, and the supernatant was aspirated. The pellet was then allowed to air dry with the tube inverted on a Kimwipe for 10 min. Sample concentration and purity were assessed *via* Qubit and Nanodrop, respectively (Thermo Fisher Scientific Nanodrop 2000 and Qubit instruments with the Qubit dsDNA BR Assay Kit [Cat #Q32853]). RNA integrity was tested using an Agilent 2100 Bioanalyzer.

### Nanopore ligation-based DNA sequencing

DNA sequencing of Nanopore replicates 1 and 2 was performed using the SQK-LSK110 Ligation sequencing kit using the manufacturer’s protocol. Genomic DNA (∼1 μg as measured by Qubit) was sequenced on an R9.4.1 (Cat # FLO-MIN106D) flow cell for 24 h, washing between samples as per manufacturer’s recommendations (EXP-WSH003).

### Nanopore direct RNA sequencing

RNA sequencing of Nanopore replicates 1 and 2 was performed using the SQK-RNA0002 Direct RNA-sequencing kit from Oxford Nanopore Technologies using 500 ng RNA. All sequencing was performed on an R9.4.1 flow cell for 24 h, washing between samples as per manufacturer’s recommendations (EXP-WSH004).

### Illumina DNA sequencing

Illumina DNA sequencing libraries were created using the Illumina DNA PCR-Free Kit with 100 ng of total input (Cat # 20041794). Illumina libraries were sequenced on a NextSeq 550 Mid Output 150 × 150 paired-end sequencing run.

### PacBio sequencing

PacBio libraries were created using the PacBio SMRTbell Express Template Prep kit 2.0 (Cat # TPK 2.0) using an input of 2 μg gDNA that was sheared with the Covaris g-TUBE to an average fragment length of 10 kb (Cat # 520079). The library was sequenced on a PacBio Sequel using a SMRT Cell 1M v3 LR at a 10 pM library loading concentration with a 2-h pre-extension time and a 20-h movie time (Cat # 101-531-000).

### Data analysis

High-quality HiFi reads were extracted from PacBio sequencing data requiring a minimum of three full passes in Cirular Consensus Sequence command (v6.0.0) (31). The HiFi reads were *de novo* assembled using HiCanu, specifying a genome size of 23 Mb. All contigs were aligned to the Py17X reference genome using minimap2 (38), and all small contigs that had <2% alignment with a 17X chromosome were filtered out. Chromosome names were assigned based on alignment to Py17X. A consensus genome was generated based on the alignment of the resulting contigs to the 17X reference genome based on the following criteria: (1) if a region was missing in the assembly, then the reference bases from the missing region were added to the consensus (such a correction was only necessary at the very end of chromosomes in the subtelomeric/telomeric sequences); (2) if overlapping contigs mismatched at a base and one of the alleles matched the reference, then the reference bases were added to the consensus; (3) if overlapping contigs mismatched at a base and there was a majority allele, then that majority allele was added to the consensus (File S1). The apicoplast genome was circularized using Circlator (v.1.5.5) (62), followed by manual correction of coordinates based on Py17X alignment. Finally, we wish to note that, even in the era of high accuracy, long-read sequencing, assembling a genome can still pose many unexpected challenges. The presence of highly variable regions or extrachromosomal DNA can preclude resolution into a single haplotype at the assembly level. Because of this, substantial effort and numerous postprocessing steps may be required to produce a representative genome. The complete workflow used in this study is provided in Figure 2.

The second assembly approach utilized both Nanopore and Illumina DNA-seq reads. All Nanopore-based raw reads were first analyzed using Nanoplot (v1.33.0) (32) for quality control purposes. Nanopore reads were assembled using Flye (v2.9) (33) software and were scaffolded with Ragtag.py using the Py17X genome as a reference (34). The resulting assembly was polished in a multistep approach. First, the assembly was polished using nextPolish (v1.3.1) (35). The homopolymer and long indel errors that were still present were corrected in the second step. For these, 150 base pair reads were simulated from the Py17X genome and mapped to the polished assembly in step 1 using bwa mem (63). Variants were called with freebayes (v 1.2.0) (64), and a consensus was created with bcftools (v 1.15) (65), resulting in a second round of polished assembly. To further correct errors, we mapped the Py17XNL Illumina genomic DNA to the resulting assembly, called variants, and generated a consensus. This resulted in a third-round polished assembly. Overlapping contigs were merged, and the apicoplast sequences were circularized. The low-complexity regions and tandem repeats in both assemblies were soft masked using the tantan program (66). Assembly reports were generated to compare Nanopore-based and PacBio-based genomes using the Quast program. The variants between Py17X and Py17XNL genomes were obtained from the minimap2 (v2.18) (38) whole genome alignment using paftools.js. The variants were annotated, and variant effects were obtained using SnpEff (v.5.1d) (67). The assembly completeness was assessed using BUSCO (46). For this assessment, the *Plasmodium* lineage database (plasmodium_odb10), which contained 3642 sequences from 23 *Plasmodium* species, was searched to check for the presence and completeness of the single-copy marker genes.

To create gene models and assign gene names, Braker2 (v2.1.6) (44) was first used to predict genes, which was followed with the use of reciprocal blastp. Two sets of gene models were generated for this assembly. For the first set, Nanopore dRNA-seq reads were mapped to the assembled genome with minimap2 (v2.18) (38). Gene names were assigned by a reciprocal blast of the predicted proteins against Py17X proteins. For the second set of gene-model predictions, both Nanopore dRNA-seq and Illumina RNA-seq datasets were used. Illumina RNA-seq reads were mapped to the assembled genome using Hisat2 (v.2.2.1) (43) and were merged with Nanopore dRNA-seq alignments, which were then used for Braker2 gene-model prediction. In addition, Prokka (v 1.14.6) (68) was used to make gene predictions in mitochondria and apicoplast that were missed by Braker2. Before implementing this strategy with our Py17XNL_2 genome, Prokka’s utility in predicting organellar genes was verified by calling genes in the Py17X organellar genomes. Finally, tRNAs were predicted using tRNASCAN-SE (v.2.0.9.) (69), and rRNAs were identified by a blast search of the assembled genome using Py17X rRNAs.

### Data availability

Datasets associated with this study are available using the following identifiers: SRA BioProject, PRJNA769959; Nanopore assembly accessions, CP086268-CP086283; PacBio assembly accessions, CP115525-CP115540. All assembly files produced in this study are provided as File S2 and will be provided to VEuPathDB/PlasmoDB for integration and community use.

## Supporting information

This article contains supporting information.

## Conflict of interest

The authors declare that they have no conflicts of interest with the contents of this article.
